# Lessons from Laparoscopic Liver Surgery: A Nine-Year Case Series

**DOI:** 10.1155/2008/458137

**Published:** 2008-08-04

**Authors:** Laura Spencer, Matthew S. Metcalfe, Andrew D. Strickland, Elisabeth J. Elsey, Gavin S. Robertson, David M. Lloyd

**Affiliations:** Department of Hepatobiliary and Laparoscopic Surgery, Leicester Royal Infirmary, Leicester University, Infirmary Square, Leicester LE1 5WW, UK

## Abstract

*Objective*. This series describes a developing experience in laparoscopic liver surgery presenting results from 40 procedures including right hemihepatectomy, left lateral lobectomy, and microwave ablation therapy. *Methods*. Forty patients undergoing laparoscopic liver surgery between September 1997 and November 2006 were included. The data set includes: operative procedure and duration, intraoperative blood loss, conversion to open operation rates, length of hospital stay, complications, mortality, histology of lesions/resection margins, and disease recurrence. *Results*. Mean age of patient: 59 years, 17/40 male, 23/40 female, 23/40 of lesions were benign, and 17/40 malignant. Operations included: laparoscopic anatomical resections *n* = 15, nonanatomical resections *n* = 11, microwave ablations *n* = 8 and deroofing of cysts *n* = 7. Median anaesthetic time: 120 minutes (range 40–240), mean blood loss 78 mL and 1/40 conversions to open. Median resection margins were 10 mm (range 1–14) and median length of stay 3 days (range 1–10). Operative and 30-day mortality were zero with no local disease recurrence. *Conclusion*. Laparoscopic liver surgery appears safe and effective and is associated with reduced hospital stay. Larger studies are required to confirm it is oncologically sound.

## 1. INTRODUCTION

Experience in laparoscopic liver surgery is growing worldwide. This single centre series describes 40 laparoscopic liver procedures carried out between September 1997 and November 2006. Included are both anatomical and nonanatomical laparoscopic resections for both malignant and benign tumours and novel use of the microwave ablation probe on hepatocellular carcinoma and other benign lesions. Operating times, resection margins, length of hospital stay, amount of blood loss, and technical aspects of laparoscopic liver surgery are discussed.

## 2. PATIENTS AND METHODS

Forty patients undergoing laparoscopic liver surgery by two surgeons were identified from operating diaries between September 1997 and November 2006. Information was gathered on a standard proforma retrospectively from operating notes, anaesthetic charts, histology reports, hospital notes, hospital computer systems, general practitioner records, and direct observation of procedures.

 No patient was excluded and patients were selected for laparoscopic surgery as having a lesion which was technically possible to resect or treat laparoscopically whilst leaving an adequate resection margin where applicable. In the case of patients with hepatic tumours, a standard multidisciplinary team approach in regards to preoperative staging, treatment, and selection was adopted. Patients selected for microwave ablation included: patients with HCC who would not withstand formal liver resection due to poor hepatic function, one patient undergoing laparoscopic left lateral lobectomy for a suspicious lesion (at operation suspected to be FNH), and a similar suspicious lesion in segment 4a which was biopsied and then microwaved—this prevented a major hemihepatectomy in a young patient for benign disease, an inaccessible haemangioma in segment 7, and a metastasis from a parathyroid carcinoma in segment 4 preventing a major resection in a frail patient.

Laparoscopic liver surgery was carried with most patients in a supine low Lloyd-Davies position, some right sided lesions were accessed by positioning the patient on their left side. One surgeon (DML) used an open approach for obtaining a pneumoperitoneum for all candidates including those who had had prior midline laparotomies. The second surgeon (GSR) preferred to use a Veress needle inserted at Palmer's point (just below the left costal margin in the midclavicular line), to insufflate the abdomen and introduced a 5 mm laparoscope in the left upper quadrant to avoid any adhesions whilst introducing the umbilical trocar in those with prior midline laparotomies [1]. In patients with no prior surgery, an open approach was used by GSR. A thirty-degree laparoscope was used in all instances. Carbon dioxide pressures were maintained between 8–12 mmHg. In general 5 ports including two 10/12 ports to interchange the cavitronic ultrasound surgical aspirator (CUSA) (Valleylab Tyco Healthcare, Boulder, Colo, USA) and camera were used. No hand assistance was used. Resections were carried out using the harmonic scalpel (Ethicon Endosurgery, Cincinnati, Ohio, USA) or argon beam coagulator (Valleylab Tyco Healthcare) to cut through the capsule, and CUSA for parenchymal dissection in conjunction with an Allports vascular staple gun (Ethicon Endosurgery), or a multiple clip applier for clipping vessels. The harmonic scalpel uses ultrasonic energy vibrating at 55 500 Hz to allow precise cutting and focussed coaptive coagulation allowing preservation of important structures such as bile ducts and larger blood vessels. Coaptive coagulation requires a lower temperature (50°C to 100°C) than electrocautery or lasers to denature proteins in the tissues forming a haemostatic protein coagulum which seals smaller blood vessels, when the effect is prolonged, secondary heat is produced and can seal larger vessels useful for fast effective haemostasis in laparoscopic hepatectomy. The argon beam coagulator is useful in laparoscopic hepatectomy as it may coagulate a large area such as the cut surface of the liver after resection using lower temperatures (50°C to 104°C) than standard electrocautery, thus minimising eschar and damage to important structures such as bile ducts and large vessels.

A single Pringle manoeuvre was applied for 30 minutes during the laparoscopic right hemihepatectomy, no other resections required a Pringle manoeuvre and no outflow control was necessary in any procedure. Intraoperative ultrasound (BK Medical, Herlev, Denmark) was used to assess extent of malignant tumours in all cases. At the end of the resection, fibrin glue (Johnson and Johnson, Cincinnati, OH, USA) was used to seal the resection site. All tumours were sealed in a retrieval pouch (Espiner Medical Limited retrieval products, Bristol, UK) or an endocatch (Autosutures, Tyco Healthcare, Hampshire, UK) before removal from the abdomen. For microwave ablation (Microsulis Medicial Limited, Hampshire, UK) the 5 mm probe was inserted through a laparoscopic port and the ceramic tip of the probe inserted directly into the lesion, microwaves were then radiated from the tip. Ablation progress was monitored by real-time laparoscopic intraoperative ultrasound. At the end of the operation, a tube drain is placed in the liver bed to monitor any postoperative bile leaks. When deroofing large liver and bile duct cysts a portion of omentum is sutured into the defect to prevent bile leaks and a tube drain is again left in situ for postoperative monitoring.

## 3. RESULTS AND DISCUSSION

Forty-three procedures including 41 laparoscopic liver procedures on 40 patients were performed between September 1997 and November 2006. The mean age of patient at operation was 59 years, 17/40 of patients were male and 23/40 were female. The median American Society of Anaesthesiologist's (ASA) score was 2.

Malignant lesions resected were colorectal metastases. Benign lesions treated included haemangiomas, liver cell adenoma, focal nodular hyperplasia, and deroofing of liver cysts and a bile duct cyst. Microwave ablation was utilised for hepatocellular carcinoma, haemangioma, metastatic parathyroid carcinoma, and focal nodular hyperplasia. Three operations incorporated multiple procedures these included a 74-year-old frail male patient who had a right inguinal hernia repair immediately before proceeding to a planned segment 5 resection of a colorectal metastasis. This negated the requirement for a second anaesthetic. A 73-year-old male who had a wedge excision of a haemangioma in segment 5 and microwave ablation of a second highly vascular haemangioma in segment 6, minimising the amount of tissue resected for benign disease. A 34-year-old female undergoing laparoscopic left lateral lobectomy for a suspicious lesion thought to be FNH, with a similar suspicious lesion found in segment 4a, intraoperative frozen section confirmed this lesion also to be FNH and a successful microwave ablation was performed preventing a formal hemihepatectomy in a young patient with benign disease.

The operations included right hemihepatectomy *n* = 1, (a second right hemihepatectomy was aborted due to widespread peritoneal disease following an exploratory laparoscopy *n* = 1), left lateral lobectomy *n* = 4, bisegmentectomy *n* = 2, segmetectomy *n* = 8, wedge resection *n* = 11, microwave ablation *n* = 8, and deroofing of liver and bile duct cysts *n* = 7, right inguinal hernia repair *n* = 1. Table 1 describes the demographic, intraoperative and postoperative details of all the patients.

Median operating time for the entire group of patients as judged by length of time under general anaesthetic in theatre was 120 minutes (range 40–240 minutes). Mean blood loss was 78 mL and only one patient required transfusion. Conversion to open operation was carried out in 1 out of 40 patients; this was a segment 5 resection for a colorectal metastasis and was due to CUSA failure. There was no perioperative mortality and no 30-day mortality. Median total length of stay in hospital was 3 days. No bile leaks were experienced and there were no other complications.

Median operating time excluding patients having only cyst deroofing or microwave ablation, and excluding the patient who was converted to open hepatectomy and the patient having a failed right hemihepatectomy due to the presence of peritoneal metastasis, was unchanged at 120 minutes (range 60–240 minutes), mean blood loss was 104 mL, and median length of stay remained 3 days for this group. This data is described in Table 2.

Median resection margins given by the pathologist were 10 mm (range 1–45 mm), where actual resection margins were not given malignant lesions were described as completely excised (CE). Of the 17 malignant tumours, one patient has died of coronary artery disease and one patient who had a segment 5 resection of colorectal liver metastasis has developed lung metastases. No patient has had locally recurrent disease.

## 4. DISCUSSION

The benefits and indications for laparoscopic liver surgery have yet to be fully established. The first reported laparoscopic liver resection was undertaken in 1991 for a benign lesion [2]. Since then data from a recent review suggests that most laparoscopic resections have been nonanatomical or simple wedge resections or cyst fenestration and deroofing for liver cysts [3]. Left lateral lobectomy (Couinaud segments 2 and 3) has been carried out and also more rarely formal left or right hemihepatectomy [4]. Approximately 70% of resections have been carried out for benign lesions and 30% malignant lesions [5]. The largest series reports 100 resections [6].

Laparoscopic liver surgery is a developing field and our experience and learning curve together with a discussion pertaining to indications, safety, and efficacy of laparoscopic hepatic resection and technical aspects of the surgery is presented.

**Figure 1:**
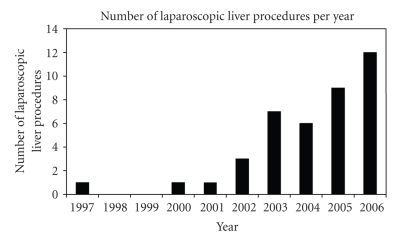
Graph showing the number of laparoscopic liver procedures per year 1997-2006.

The first laparoscopic liver resection at our institution was a left lateral lobectomy in 1997 for focal nodular hyperplasia. Since then the number of hepatic resections carried out laparoscopically has increased year on year see Figure 1.

Lesions selected for laparoscopic resection at our institution are in the easily accessible segments: 2, 3, 4a, 5, and 6 well away from the major hepatic veins. These lesions are often visible on the liver surface and well characterised by saggital and coronal magnetic resonance images. Initially only benign lesions were resected due to concerns regarding oncological adequacy of laparoscopic resection margins and the risk of tumour seeding in the port sites. The publication of the conventional versus laparoscopic-assisted surgery in patients with colorectal cancer (CLASICC) trial verified that there were no significant differences between open surgery and laparoscopic-assisted colorectal tumour excision with respect to tumour and nodal status, therefore, laparoscopic surgery in relation to excision of malignant hepatic tumours seemed justified [7].

Microwave ablation has been under investigation by the authors for the treatment of liver tumours in both animal models and human patients [8–10]. The indications for the use of microwave ablation are, as yet, not clearly defined though patients with HCC who could not withstand a formal hepatectomy due to loss of hepatic tissue, symptomatic benign tumours (excluding liver cell adenoma), unusual single liver tumours, and patients with tumour recurrence in a liver that has previously undergone a resection are all potential candidates and have been described in this series.

Controversy exists as to the adequacy of tumour excision and resection margin. One large seriesreports median resection margins of 5 and 10 mm for colorectal metastasis and HCC, respectively [4]. Usage of the intraoperative ultrasound probe has been shown by Gigot et al. [11] to improve adequacy of resection margin. The intraoperative ultrasound probe is therefore used routinely in our establishment when resecting malignant tumours.

There are currently no randomised controlled trials comparing open and laparoscopic hepatectomy but several small nonrandomised comparative trials have suggested that there is no difference in adequacy of resection margin between open and laparoscopic hepatectomy [12–15]. The median resection margin for malignant tumours in this study is 10 mm and there has been no local recurrence. Recent studies have now suggested that the width of a negative resection margin does not affect survival, recurrence risk, or site of recurrence for colorectal metastasis [16]. It is of note that anatomical resections for colorectal metastases have also not been shown to be superior to wedge resections as long as the tumour is clear at the resection margins [17].

Abdominal wall metastasis at port sites has been reported after laparoscopic surgery for malignant tumours [18]. Recent data has shown that there is no difference in wound-site tumour recurrence between open and laparoscopic colorectal surgery for if conventional oncological techniques such as no touch technique and sealing resected specimens in retrieval pouches before removal are employed, rates should be less than 1% [19]. No wound site recurrence has been experienced in this series to date.

Some surgeons have raised concerns for the possibility of gas embolism due to transection of low pressure veins under pneumoperitoneum. This has led some centres to adopt a gasless laparoscopy technique, whereby intra-abdominal space is created by abdominal suspension techniques [20]. Others have used a lower pressure carbon dioxide pneumoperitoneum, maintained between 10–12 mmHg [4, 12].

Usage of the argon beam coagulator in both laparoscopic cholecystectomy and laparoscopic liver surgery has been associated with a clinical diagnosis of venous gas embolism in several case reports [21–23]. A recent review suggests that the incidence of clinical gas embolism in 700 laparoscopic liver procedures carried out worldwide is reportedly 0.3% [5]. It is, however, of interest that following laparoscopic cholescystectomy intravascular gas bubbles have been routinely observed using transoesophageal echocardiography; this does not seem to correlate with the clinical picture of gas embolism [24].

An experimental study assessing the usage of argon-enhanced coagulation of cut sections of liver in a porcine model, whereby gas emboli were detected using a Doppler flow cuff around the caudal vena cava, demonstrated that the number of gas emboli produced increased with increasing gas flow rates but was not affected by coagulation power. This study recommended that surgeons and anaesthetists should be aware that there is a potential to develop a clinically important gas embolism and that adequate precautions should be taken to prevent this such as selecting a low flow setting on the argon beam coagulator and adequate venting of the abdomen through chimneys in laparoscopic ports to maintain safe pressures of between 8–12 mmHg [25]. These measures have been adopted at our institution where argon gas flow is set to 4 L/min (low flow) on the laparoscopic setting which incorporates an alarm if intra-abdominal pressures exceed preselected limits—12 mmHg for laparoscopic hepatectomy which alerts the surgeon who can open the port chimney to vent the abdomen. No clinical signs of gas embolism have been observed in association with the argon beam coagulator. Hepatectomy can be associated with severe haemorrhage and a major concern to some is the threat of uncontrollable haemorrhage with minimal access to the abdomen. This series presents an extremely low operative blood loss rate with a mean blood loss of 78 mL. It is worthwhile noting that where laparoscopic liver resections are converted to open procedures it is often due to bleeding.

In general, some studies comparing laparoscopic versus open procedures have demonstrated that the laparoscopic approach may well be associated with less blood loss than open equivalents [14, 26]. This observation may be due to the tamponading effect of a pneumoperitoneum and the anaesthetists at our institution raise the central venous pressure to between 7–10 mmHg whilst the surgeons decrease the carbon dioxide insufflation pressure at the end of all laparoscopic liver procedures in order to assess safely for haemostasis. Additional anaesthetic considerations pertaining to laparoscopic liver surgery include initially running a lower central venous pressure of 1-2 mmHg to decrease bleeding and placement of a central line for central venous pressure monitoring, but an epidural is not required as postoperative analgesia requirements are usually confined to oral preparations.

The final consideration for safe laparoscopic surgery pertains to technical ability and experience of the surgeon. It is recommended by the specialist advisory committee to the National Institute of Clinical Excellence that laparoscopic liver resections should only be carried out in specialist centres and by those with advanced skills in hepatic resection and laparoscopy [27]. It is also apparent that the lesions most suitable for laparoscopic resection are those situated in the left lateral and right anterior segments away from the main hepatic veins this in turn reduces the risk of serious haemorrhage.

In conclusion this series confirms evidence already published in support of laparoscopic liver resections. Patients experience a shorter hospital stay, minimal blood loss, shorter operation time, and short-term data indicates equivalent oncological results when compared with conventional open hepatectomy. Randomised controlled trials assessing the oncological efficacy of laparoscopic and open hepatectomy for selected lesions are indicated at this stage.

## Figures and Tables

**Table 1:** Demographic, intraoperative, and postoperative details of patients undergoing laparoscopic liver surgery.

Patient	Age	Sex	Procedure	Histology	Operation time	Blood loss	Resection margin	Length of	Complications
		(M/F)			(minutes)	(mL)	(mm)	stay (days)	
1	63	M	RHH	CRM	240	1500	1	4	Nil
2	60	M	WES3	HMG	240	0	CE	2	Nil
3	51	F	WES5	LCA	120	0	CE	3	Nil
4	68	F	S5/6R	CRM	240	0	CE	5	Nil
5	74	F	S4R	CRM	180	0	22	3	Nil
6	69	M	S6R	CRM	105	0	5	3	Nil
7	74	M	S5R+RIH	CRM	180	0	CE	7	Nil
8	43	F	S2R	FNH	120	0	CE	3	Nil
9	75	M	S3R	CRM	240	0	10	3	Nil
10	82	M	LLB	CRM	180	500	CE	4	Nil
11	77	M	MAS4/8	HCC	150	0	CE	6	Nil
12	45	F	WES6	HMG	180	0	45	6	Nil
13	64	M	LLB	CRM	240	0	10	4	Nil
14	73	M	S6R	CRM	240	600	4	6	Nil
15	73	M	WES6, MAS5	HMG	90	0	CE	4	Nil
16	70	M	MAS4a	HCC	90	0	N/A	7	Nil
17	69	M	S5R	CRM	210	500	CE	10	Nil
18	57	F	WES5	HMG	120	0	CE	2	Convert to open due to CUSA failure
19	36	F	LLB	FNH	120	0	CE	3	Nil
20	34	F	LLB,MAS4	FNH	120	0	CE	3	Nil
21	55	M	MAS7	HMG	90	0	N/A	2	Nil
22	58	F	WES3 SOC	LC	90	0	CE	2	Nil
23	38	M	EL: (Planned RHH aborted)	CRM Peritoneal deposits	90	0	N/A	2	Nil
24	51	M	WES2/3	HMG	120	0	CE	3	Nil
25	47	F	WE2/3	Angiomyo-leiyoma	60	0	CE	2	Nil
26	52	F	WES3	Mucinous cystaden-oma	60	0	CE	1	Nil
27	62	F	LDBDC, SOC	Biliary cystaden-oma	40	0	N/A	2	Nil
28	41	F	LDLC	LC	40	0	N/A	2	Nil
29	73	F	LDLC	LC	60	0	N/A	1	Nil
30	57	F	LDBDC, SOC	BDC	60	0	N/A	2	Nil
31	56	F	LDLC, SOC	LC	60	0	N/A	1	Nil
32	84	M	S5R	CRM	60	10	CE	1	Nil
33	42	F	LDPCL	LCPCL	30	0	N/A	3	Nil
34	79	M	LDPCL	LCPCL	60	0	N/A	3	Nil
35	71	F	MAS4	HCC	40	0	N/A	2	Nil
36	32	F	WES5/6	FNH	120	0	30	3	Nil
37	43	F	MAS4	Parathyro-ID carcinoma metastasis	60	0	N/A	1	Nil
38	51	F	WES6	HMG	120	0	20	3	Nil
39	72	F	S4/S6R	CRM	200	0	4	3	Nil
40	34	F	MA	FNH	130	0	N/A	2	Nil

**Table 2:** 

	All patients (including resections, ablations, and cyst deroofings) *N* = 40	Patients undergoing only laparoscopic hepatectomy *N* = 25
Median operation time (minutes)	120	120
Mean blood loss (mL)	78	104
Median length of stay (days)	3	3
